# Schools in Medicine

**Published:** 1887-12

**Authors:** 


### SCHOOLS IN MEDICINE.

An editorial in the *New York Medical Times* for November is very square footed upon the two more pretentious schools of medicine, using the following language :

"The two greater so-called schools in medicine seem to be failing entirely in their duties to each other and to the public! There has been, and is now, a regular system of boycott going on in both of them toward such of their associates, as dare to have opinions respecting eithics at variance with what these schools choose to dogmatically lay down, as guides in their relations with one another!

In practice, the great majority of both these bodies are in substantial accord, so far as the use of therapeutic means is concerned, that is to say, all assert their willingness to adopt that means which will best subserve the interests of their patients!

In the Old School class, many seem afraid to employ small doses, even when confident of their superiority, for fear of the homœopathic designation, and the homœopathic class fights shy of the large dose, even when absolutely called for, for fear that they may be termed allopaths! Both these sects are thus doing themselves and the public an injury, and all on account of the sectarian designation.

How much better it would be for both of these parties, to come out honestly and squarely and admit that there are occasions when small doses would be best, and that there are other instances when the larger, even the largest, may be required, depending entirely upon the effects to be obtained, and the mode of selection will be easily admitted, as it will be determined also by the end desired. The Old School undoubtedly occupies the higher relative plane in the controversy by being free from sectarian designation, but still, as a body, it has a great responsibility at this juncture, and one that it should not shirk nor attempt to temporize with.

It is highly improper to use the expression 'practicing both ways,' and if there were no sectarian designation, there would be no opportunity to make such a statement !

A celebrated divine once said, regarding the Christian science craze of which he was supposed to be a supporter, after criticising some of their methods, that he was not on the fence respecting it, but on both sides of the fence ! In reply to the query of an auditor as to how a man could face both ways, he promptly replied that he 'could look all around ! '

So it should be respecting medicine, we ought to occupy a position in which we can *see all around*, and to do this successfully, we must not be cramped, but free to examine all things and to hold fast to such as are found to be unquestionably good ! "

